# No clinically significant pharmacokinetic interactions between dolutegravir and daclatasvir in healthy adult subjects

**DOI:** 10.1186/s12879-016-1629-5

**Published:** 2016-07-22

**Authors:** Lisa L. Ross, Ivy H. Song, Niki Arya, Mike Choukour, Jian Zong, Shu-Pang Huang, Timothy Eley, Brian Wynne, Ann M. Buchanan

**Affiliations:** ViiV Healthcare, Research Triangle Park, NC USA; GlaxoSmithKline, Research Triangle Park, NC USA; Parexel, Research Triangle Park, NC USA; Pharstat, Research Triangle Park, NC USA; Bristol-Myers Squibb, Princeton, NJ USA

**Keywords:** Adult medicine, Drug interactions, Infectious disease, Interactions, Pharmacokinetics

## Abstract

**Background:**

Daclatasvir (DCV) is an NS5A replication complex inhibitor recently approved for chronic hepatitis C virus treatment.

**Methods:**

To assess drug interactions between the HIV integrase strand transfer inhibitor dolutegravir (DTG) and DCV, subjects were randomized into 1 of 2 sequences in an open-label, 3-period, crossover study. Subjects received either DTG 50 mg once daily or DCV 60 mg once daily for 5 days in periods 1 and 2 and DTG 50 mg plus DCV 60 mg once daily for 5 days in period 3, with no washout between periods 2 and 3. Between periods 1 and 2, there was a washout period of at least 7 days.

**Results:**

The geometric least-squares mean ratios (90 % confidence intervals) of DCV area under the concentration-time curve over a dosing interval (AUC_0-τ_), maximum observed concentration (C_max_), and concentration at the end of the dosing interval (C_τ_) were 0.978 (0.831–1.15), 1.03 (0.843–1.25), and 1.06 (0.876–1.29), respectively, when DCV was administered with DTG compared with DCV alone. Compared with DTG alone, coadministration of DTG with DCV increased DTG AUC_0-τ_, C_max_, and C_τ_ by approximately 33, 29, and 45 %, respectively.

**Conclusions:**

DCV plasma exposure was not meaningfully affected by DTG. Coadministration of DTG with DCV resulted in slight increases in DTG AUC_0-τ_, C_max_, and C_τ_. Accumulated safety and tolerability data in humans receiving DTG to date suggests this effect is not considered clinically significant. DTG and DCV can be coadministered without dose adjustment.

**Trial registration:**

Registered on March 6, 2014 with ClinicalTrials.gov; registration number: NCT02082808 and as Study ID: 201102 on the ViiV Clinical Study Registry.

## Background

HIV and hepatitis C virus (HCV) are both blood-borne viruses, with transmission in adults occurring primarily through injection drug use or sexual contact. Both HIV and HCV can also be transmitted from mother to child during birth or through breastfeeding. Viral infection can also occur in rare circumstances when virally infected bodily fluids come into contact with a noninfected person’s bloodstream, mucous membranes, or damaged tissue such as through use of contaminated needles or other medical equipment, transfusion of virally contaminated blood or blood products, or various other routes [1, 2]. Coinfection with both HIV and HCV is not uncommon because of shared transmission modes. Globally, approximately 7 million people are thought to be coinfected with both HIV and HCV and, in the United States, about one-quarter to one-third of HIV-infected individuals are estimated to be coinfected with HCV [3]. Infection with HCV can result in long-term illness and death, and viral hepatitis progresses faster and causes more liver-related health problems among people living with HIV than among those without HIV infection. Although combination antiretroviral therapy has extended the life expectancy of people with HIV, liver disease has become the leading cause of non-AIDS-related deaths in this population, and HIV-infected people coinfected with HCV are at increased risk for serious, life-threatening complications [3–5]. Treatment for HCV infection has made remarkable progress in the last several years, from treatments with relatively low cure rates that typically included pegylated interferon alfa in combination with ribavirin, to all-oral, direct-acting antiviral regimens that produce sustained viral responses with cure rates exceeding 90 % for many patient populations [5].

Dolutegravir (DTG, Tivicay^®^, ViiV Healthcare, Research Triangle Park, NC) is an HIV-1 integrase strand transfer inhibitor approved by the Food and Drug Administration and the European Medicines Agency for the treatment of HIV-1 infection in a broad patient population [6, 7]. Daclatasvir (DCV, Daklinza^™^, Bristol-Myers Squibb, Princeton, NJ) is an inhibitor of the HCV nonstructural protein NS5A and has been approved by the European Medicines Agency for use in combination with other medicinal products across genotypes 1, 3, and 4 for the treatment of chronic HCV infection in adults and by the Food and Drug Administration for the treatment of chronic HCV genotype 3 infection in adults [8, 9]. Because DTG and DCV may be concomitantly administered in subjects coinfected with HIV-1 and HCV, a drug interaction study between DTG and DCV was warranted to evaluate the pharmacokinetics (PK) of both drugs, as well as patient safety and drug tolerability when these drugs are coadministered.

## Methods

### Study design and subjects

This was an open-label, 3-period, crossover study conducted at a single-center in healthy adult subjects. The study protocol, amendments, and consent forms were reviewed and approved prior to study initiation by the study site’s institutional review board (Quintiles Early Clinical Development, Overland Park, KS, USA), and all subjects provided their written consent prior to initiation of any study-specific procedures. The study was conducted in a manner consistent with good clinical practices and local regulatory requirements and in accordance with the ethical standards of the Declaration of Helsinki and its amendments,

Adult men and women ranging from 18 to 65 years of age with a body mass index ranging from 18.5 to 31.0 kg/m^2^ (inclusive) and no clinically significant abnormalities on the basis of physical examination, laboratory testing, 12-lead electrocardiogram, and medical history were eligible for study inclusion. Eligible female subjects had negative results on human chorionic gonadotropin pregnancy tests. Male or female subjects who were not surgically sterile/postmenopausal agreed to use nonhormonal contraceptive methods, including abstinence, an intrauterine device, or 2 forms of barrier contraception. Excluded subjects were those with a preexisting condition that interfered with normal gastrointestinal motility or anatomy; as well as subjects with hepatic dysfunction or renal dysfunction, or both that could have interfered with the study drugs’ absorption, metabolism, or excretion. Subjects were excluded if they had an average weekly alcoholic beverage intake of more than 14 drinks for males or more than 7 drinks for females within 6 months of the study start. Exclusion criteria also included the regular use of tobacco- or nicotine-containing products within 6 months prior to screening; or a positive test for HCV antibody, hepatitis B surface antigen, or HIV antibody. Eligible subjects were prohibited from ingesting any prescription or nonprescription drugs, including herbal and dietary supplements and vitamins within 30 days, 5 half-lives, or twice the duration of the biological effect of the investigational product, whichever was longer, before the first dose of study medication.

Twelve healthy male or female subjects were enrolled to provide data from at least 10 evaluable subjects. The sample size of 12 to obtain 10 evaluable subjects was chosen based on an expected withdrawal rate of approximately 10 % and the within-subject variability of DTG. Within-subject variability of DCV, based upon historical data, is lower than that of DTG. Using a within-subject variability (CVw) of 30 % and a sample size of 10 evaluable subjects, it was estimated that the precision for the treatment comparison would be within 25.8 % of the point estimate for the concentration-time curve over the dosing interval (AUC_[0-τ]_), C_max_, and C_τ_. If the point estimate of the ratio of geometric means was 1, then the 90 % confidence interval would be approximately 0.79 to 1.26. Subjects were randomized into 1 of 2 treatment sequences (*n* = 6 in each sequence) according to a randomization schedule. Study medications were DTG as a 50-mg tablet given once daily and DCV as a 60-mg tablet given once daily. Subjects in sequence 1 received DTG for 5 days in period 1 (treatment A), followed by DCV for 5 days in period 2 (treatment B), and finally DTG plus DCV for 5 days in period 3 (treatment C). Those in sequence 2 received DCV (treatment B) for 5 days in period 1, followed by DTG (treatment A) for 5 days in period 2, and then DTG plus DCV for 5 days in period 3 (treatment C). All doses of study drug were ingested under fasting conditions. Between period 1 and period 2, there was a washout period of at least 7 days. There was no washout period between period 2 and period 3. Day 1 of period 3 started the day after the last day in period 2. Periods 1, 2, and 3 were conducted on an inpatient setting. Within 7 to 14 days after the last dose of study drug was taken, a follow-up visit was conducted.

The safety evaluations performed during the study included clinical laboratory tests (hematology, serum chemistry, and urinalysis), vital sign monitoring, and physical examinations. Electrocardiograms were performed at screening. Throughout the entire treatment phase and at the follow-up evaluation, there was close monitoring for all adverse events (AEs).

### Pharmacokinetic assessments

Blood samples were collected (2 mL per collection) at predose (within 15 min prior to dosing) and at 1, 2, 3, 4, 8, 12, and 24 h postdose on day 5 in period 1 or 2 and in period 3 for the determination of plasma concentrations of DTG. Blood samples were collected for the determination of plasma concentrations of DCV using the same sample collection schedule as was used in prior drug-drug interaction studies with DCV [10], which was 4 mL per collection at predose (within 15 min prior to dosing) and at 0.5, 1, 1.5, 2, 3, 4, 6, 8, 12, 16, and 24 h postdose on day 5 in period 1 or 2 and in period 3. Blood samples were drawn into potassium ethylenediaminetetraacetic acid (K_2_ EDTA)-containing tubes via venipuncture or through a cannula and were kept chilled on ice until centrifugation. Plasma was separated by centrifugation at 4 °C within 45 min of sample collection and stored at−20 °C or below until analysis.

### Bioanalytical methods

Plasma samples were analyzed for DTG concentrations by PPD (Middleton, WI). All samples were received frozen on dry ice and in acceptable condition and stored frozen at−20 °C upon arrival. The DTG analysis was done using a validated analytical method based on protein precipitation using acetonitrile, which was followed by high-performance liquid chromatography with tandem mass spectrometry analysis using positive-ion electrospray, which was based on previously published methods [11]. This assay was validated over the DTG concentration range of 20.0 to 20,000 ng/mL using a 25-μL aliquot of K_2_ EDTA-treated plasma.

Plasma samples were analyzed for DCV concentrations by Tandem Labs (West Trenton, NJ, USA). Analysis was performed using a validated analytical method of solid phase extraction, followed by high-performance liquid chromatography with tandem mass spectrometry analysis, which was based on a previously published method [12]. The lower and higher limits of quantification were 2.00 ng/mL and 2000 ng/mL, respectively, using a 100-μL aliquot of K_2_ EDTA-treated plasma.

Quality control (QC) samples containing 3 different analyte concentrations of DTG and DCV were analyzed with each batch of samples against separately prepared calibration standards that were stored under the same conditions as study samples. Quality control results met acceptance criteria; ≤33 % of the quality control results were to deviate from the nominal concentration by >15 %, with ≥50 % of the quality control results acceptable at each concentration. The calibration standard coefficient of variance (CV) for DTG was ≤6.7 % with a difference from theoretical of ≤4.9 %, and the interassay CV per run was ≤5.8 % with a difference from theoretical of ≤3.2 %. The between-run CV for DCV was ≤2.2 %, and the within-run CV was ≤2.0 % with a mean deviation from nominal concentration of ±3.6 %.

### Pharmacokinetic data analysis

Plasma DTG and DCV concentration-time data were analyzed by noncompartmental methods using Phoenix WinNonlin version 6.3 (Pharsight Corporation, St. Louis, MO). Pharmacokinetic parameter calculations were based on the actual sampling times recorded during the study. Pharmacokinetic parameters that were determined included maximum observed concentration (C_max_), concentration at the end of the dosing interval (C_τ_), AUC_0-τ_, apparent clearance following oral dosing (CL/F), and terminal phase half-life (t_1/2_).

### Statistical analysis

Statistical analysis was performed on the log-transformed plasma PK parameters. Point estimates and their associated 90 % confidence intervals (CIs) were constructed for the differences between test and reference treatments. Dolutegravir (treatment A) or DCV (treatment B), when given alone under fasted conditions, was considered to be the reference treatment. The test treatments were DTG coadministered with DCV (treatment C) under fasted conditions. The point estimates and their associated 90 % CIs were back-transformed to provide the ratios of geometric least-squares means and associated 90 % CIs for test/reference for the PK parameters AUC_0-τ_, C_τ_, C_max_, CL/F, and t_1/2_. Dolutegravir and DCV PK were analyzed separately using SAS (SAS Institute, Inc, Cary, NC).

## Results

### Subject demographics and accountability

A total of 12 subjects (6 in each sequence) were enrolled in the study, and all subjects completed study treatments and planned DTG and DCV PK sampling. One subject was lost to follow-up. There were no subjects who were prematurely withdrawn from the study due to AEs. Subjects had an overall mean age and body mass index of 32.3 years (standard deviation [SD]: 10.1) and 24.0 kg/m^2^ (SD: 3.0), respectively. The majority were male (75 %), and white and African-American subjects each accounted for 50 % of the total.

### Safety evaluation

Dolutegravir alone (50 mg once daily), DCV (60 mg once daily), and DTG plus DCV (50 mg once daily and 60 mg once daily, respectively) were well tolerated. No pattern of AE frequency was seen with respect to treatment, and none of the AEs were grade 2 or greater in severity or were considered to be drug-related. Two AEs were reported by subjects when taking DTG alone, 1 AE was reported by a subject taking DCV alone, and 2 AEs were reported when DTG and DCV were taken concurrently. Only 1 AE, headache, was reported more than once and it was reported 3 times, once with each treatment. There were no consistent, treatment-related, or clinically significant changes in median or mean clinical chemistry or hematology values observed in this study, and no clinically significant changes in electrocardiogram results or vital signs. After period 3 had been completed and during the follow-up visit, one African-American male subject had a grade 2 glucose measurement of 8.05 mmol/L (144.9 mg/dL). This subject, on one prior measurement during period 2, day−1, had a grade 1 glucose elevation of 6.22 mmol/L (112 mg/dL) that occurred after he completed DCV dosing and before he received DTG. Attempts to contact him after follow-up for additional follow-up testing were unsuccessful, and he was considered lost to follow-up.

### Pharmacokinetics of DTG

**Fig. 1:**
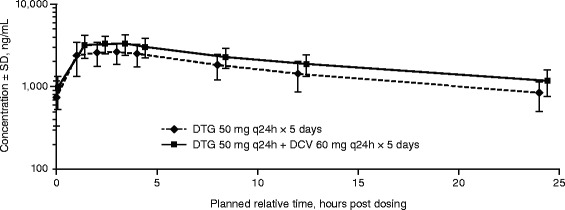
Mean plasma concentration-time profiles of dolutegravir (DTG) administered with and without daclatasvir (DCV). Abbreviations: q24h, every 24 h; SD, standard deviation

The mean plasma concentration-time profiles of DTG after administration of DTG alone and in combination with DCV are presented in Fig. 1. Coadministration of DTG 50 mg once daily with DCV 60 mg once daily increased DTG AUC_0-τ_, C_max_, and C_τ_ by 33, 29, and 45 %, respectively, compared with DTG administered alone. Dolutegravir CL/F decreased by 25 %, while the t_1/2_ increased by 17 % when coadministered with DCV compared with DTG administered alone (Table 1).

**Table 1:** Statistical comparison of DTG PK parameters when administered with and without DCV

Plasma DTG PK parameter	Geometric mean (CV%)		Geometric least-squares mean ratio (90 % CI)
	DTG alone (treatment A) (*N* = 12)	DTG + DCV (treatment C) (*N* = 12)	DTG + DCV vs DTG alone
AUC_0-τ_ (hr · μg/mL)	35.7 (34.7)	47.3 (26.3)	1.33 (1.11–1.59)
C_max_ (μg/mL)	2.65 (32.0)	3.43 (24.5)	1.29 (1.07–1.57)
C_τ_ (μg/mL)	0.771 (41.3)	1.11 (36.6)	1.45 (1.25–1.68)
CL/F (L/hr)	1.40 (34.7)	1.06 (26.3)	0.753 (0.627–0.905)
t_1/2_ (hr)	13.9 (32.8)	16.2 (32.6)	1.17 (1.01–1.35)

*Abbreviations*: *AUC*_*0-τ*_ area under the concentration-time curve over the dosing interval, *C*_*τ*_ concentration at the end of the dosing interval, *CI* confidence interval, *CL/F* apparent clearance following oral dosing, *C*_*max*_ maximum observed concentration, *DCV* daclatasvir, *DTG* dolutegravir, *PK* pharmacokinetic, *t*_*1/2*_ terminal phase half-life

Treatment A = DTG 50 mg once daily; treatment C = DTG 50 mg once daily plus DCV 60 mg once daily

### Pharmacokinetics of DCV

**Fig. 2:**
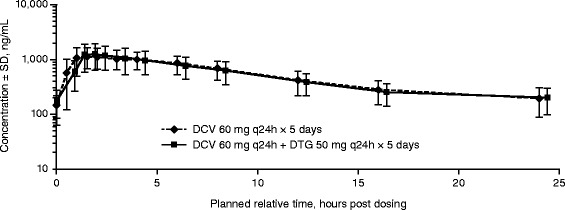
Mean plasma concentration-time profiles of daclatasvir (DCV) administered with and without dolutegravir (DTG). Abbreviations: q24h, every 24 h; SD, standard deviation

The plasma concentration-time profiles after administration of DCV alone and in combination with DTG are presented in Fig. 2. DCV exposure did not appear to be meaningfully affected by coadministration with DTG 50 mg once daily (Table 2). DCV AUC_0-τ,_ decreased by 2.2 %, C_max_ increased by 3 %, and C_τ_ increased by 6 % compared with DCV administered alone. DCV CL/F increased by 2 %, while the t_1/2_ increased by 1.8 % when coadministered with DTG compared with DCV administered alone.

**Table 2:** Statistical comparison of DCV PK parameters when administered with and without DTG

Plasma DCV PK parameter	Geometric mean (CV%)		Geometric least-squares mean ratio (90 % CI)
	DCV alone (treatment B) (*N* = 12)	DCV + DTG (treatment C) (*N* = 12)	DCV + DTG vs DCV alone
C_max_ (μg/mL)	1.19 (42.6)	1.22 (42.2)	1.03 (0.843–1.25)
AUC_0-τ_ (hr · μg/mL)	11.4 (47.9)	11.2 (41.6)	0.978 (0.831–1.15)
C_τ_ (μg/mL)	0.166 (69.3)	0.176 (53.8)	1.06 (0.876–1.29)
CL/F (L/hr)	5.25 (47.9)	5.36 (41.6)	1.02 (0.868–1.203)
t_1/2_ (hr)	8.58 (23.2)	8.42 (29.4)	0.982 (0.814–1.18)

*Abbreviations*: *AUC*_*0-τ*_ area under the concentration-time curve over the dosing interval, *C*_*τ*_ concentration at the end of the dosing interval, *CI* confidence interval, *CL/F* apparent clearance following oral dosing, *C*_*max*_ maximum observed concentration, *CV%* coefficient of variation, *DCV* daclatasvir, *DTG* dolutegravir, *PK* pharmacokinetic, *t*_*1/2*_ terminal phase half-life

Treatment B = DCV 60 mg once daily; treatment C = DTG 50 mg once daily plus DCV 60 mg once daily

## Discussion

The results from this study demonstrated that plasma exposure of DCV did not appear to be meaningfully affected when coadministered with DTG as compared with DCV administered alone. This result is consistent with the preclinical findings for DCV and DTG. Daclatasvir is a substrate of cytochrome P450 (CYP) 3A4 and the transporter P-glycoprotein (P-gp) [8, 9]. In vitro, DTG demonstrates minimal or no direct inhibition of CYP isozymes or of P-gp; and DTG is not considered an inducer of CYP3A4 [13].

Coadministration of DTG with DCV increased DTG AUC_0-τ_, C_max_, and C_τ_ by approximately 33, 29, and 45 %, respectively, compared with DTG administered alone. Dolutegravir is metabolized primarily through UDP-glucuronosyltransferase 1A1 with a minor component (~10 %) via CYP3A4 and is a substrate of P-gp and breast cancer resistance protein (BCRP) [8]. Daclatasvir is an inhibitor of some transporters, including P-gp, and BCRP [9], which provides a reasonable mechanistic explanation of the effect on DTG plasma exposure from the current study. However, the effect of DCV on increasing DTG plasma exposure is not considered clinically significant and does not confer significant safety risks as the increased DTG levels remain in the range previously observed in the dose-ranging clinical trial with HIV-1-infected subjects. In that trial, which evaluated 2, 10, and 50 mg once-daily DTG dosing, the ratios for AUC_0-τ_, C_max_, and C_τ_ after 10 days of administration were estimated to be 1.25 to 1.43, 1.23 to 1.40, and 1.27 to 1.42, respectively [14]. Similarly, in the VIKING-3 and−4 studies, integrase-resistant, HIV-infected patients received 50 mg of DTG twice daily, and predose (C0) DTG concentrations were obtained at day 8 and at weeks 4 and 24 [15, 16]. The geometric mean and between-subject CV% for the DTG C0 in VIKING-4 was 1.80 μg/ml (123 %; *n* = 27) at day 28 and 2.05 μg/ml (127 %; *n* = 24) at week 24; comparable to those observed in VIKING-3 at week 4 (1.90 μg/ml [113 %; *n* = 161]) and week 24 (2.14 μg/ml [93 %; *n* = 134]). There have been no apparent differences in safety profiles across DTG doses evaluated in phase IIb/III studies, and DTG is well tolerated in HIV-1-infected subjects, with no dose-limiting toxicity observed to date [15–23]. An upper limit of exposure associated with an increased incidence of AEs or significant clinical chemistry toxicity has not been identified. As a result, no DTG dose adjustment is necessary when it is coadministered with DCV.

## Conclusions

In conclusion, since DTG and DCV were well tolerated when given alone or in combination and no clinically significant increases in plasma exposure for either drug were observed, these results indicate that DTG and DCV can be coadministered without dose adjustment.

## Abbreviations

AE, adverse event; AUC_0-τ,_ area under the concentration-time curve over a dosing interval; BCRP, breast cancer resistance protein; C0, predose DTG concentrations; CI, confidence interval; CL/F, apparent clearance following oral dosing; C_max,_ maximum observed concentration; CV, coefficient of variance; CVw, within-subject variability; CYP, cytochrome P450; C_τ,_ concentration at the end of the dosing interval; DCV, daclatasvir; DTG, dolutegravir; HCV, hepatitis C virus; K_2_ EDTA, potassium ethylenediaminetetraacetic acid; P-gp, P-glycoprotein; PK, pharmacokinetic; QC, quality control; SD, standard deviation; t_1/2,_ terminal phase half-life
